# Enhanced proliferation of oligodendrocyte progenitor cells following retrovirus mediated Achaete-scute complex-like 1 overexpression in the postnatal cerebral cortex *in vivo*

**DOI:** 10.3389/fnins.2022.919462

**Published:** 2022-12-02

**Authors:** Chiara Galante, Nicolás Marichal, Franciele Franco Scarante, Litsa Maria Ghayad, Youran Shi, Carol Schuurmans, Benedikt Berninger, Sophie Péron

**Affiliations:** ^1^Institute of Physiological Chemistry, University Medical Center Johannes Gutenberg University, Mainz, Germany; ^2^Centre for Developmental Neurobiology, Institute of Psychiatry, Psychology & Neuroscience, King’s College London, London, United Kingdom; ^3^Department of Pharmacology, Ribeirão Preto Medical School, University of São Paulo, São Paulo, Brazil; ^4^The Francis Crick Institute, London, United Kingdom; ^5^Biological Sciences Platform, Sunnybrook Research Institute, Toronto, ON, Canada; ^6^Department of Biochemistry, University of Toronto, Toronto, ON, Canada; ^7^Department of Laboratory Medicine and Pathobiology, University of Toronto, Toronto, ON, Canada; ^8^MRC Centre for Neurodevelopmental Disorders, Institute of Psychiatry, Psychology & Neuroscience, King’s College London, London, United Kingdom; ^9^Focus Program Translational Neuroscience, Johannes Gutenberg University, Mainz, Germany

**Keywords:** astrocyte, gliogenesis, lineage reprogramming, neurogenesis, proliferation, proneural, Sox10, Ascl1

## Abstract

The proneural transcription factor Achaete-scute complex-like 1 (Ascl1) is a major regulator of neural fate decisions, implicated both in neurogenesis and oligodendrogliogenesis. Focusing on its neurogenic activity, Ascl1 has been widely used to reprogram non-neuronal cells into induced neurons. *In vitro*, Ascl1 induces efficient reprogramming of proliferative astroglia from the early postnatal cerebral cortex into interneuron-like cells. Here, we examined whether Ascl1 can similarly induce neuronal reprogramming of glia undergoing proliferation in the postnatal mouse cerebral cortex *in vivo*. Toward this goal, we targeted cortical glia during the peak of proliferative expansion (i.e., postnatal day 5) by injecting a retrovirus encoding for Ascl1 into the mouse cerebral cortex. In contrast to the efficient reprogramming observed *in vitro*, *in vivo* Ascl1-transduced glial cells were converted into doublecortin-immunoreactive neurons only with very low efficiency. However, we noted a drastic shift in the relative number of retrovirus-transduced Sox10-positive oligodendrocyte progenitor cells (OPCs) as compared to glial fibrillary acidic protein (GFAP)-positive astrocytes. Genetic fate mapping demonstrated that this increase in OPCs was not due to Ascl1-mediated astrocyte-to-OPC fate conversion. Rather, EdU incorporation experiments revealed that Ascl1 caused a selective increase in proliferative activity of OPCs, but not astrocytes. Our data indicate that rather than inducing neuronal reprogramming of glia in the early postnatal cortex, Ascl1 is a selective enhancer of OPC proliferation.

## Introduction

The postnatal mammalian brain is largely devoid of persistent neurogenesis, except from specialized niches such as the subependymal zone of the lateral ventricle and the subgranular zone of the dentate gyrus (Denoth-Lippuner and Jessberger, 2021). In all other brain regions, neurons lost due to disease or injury cannot be replaced, resulting in irreversible circuit dysfunction and functional impairments. Harnessing the neurogenic potential of glia to produce new neurons by direct lineage reprogramming has emerged as an approach for potential repair of diseased circuits in non-neurogenic brain areas such as the cerebral cortex (Peron and Berninger, 2015).

The basic helix-loop-helix (bHLH) transcription factor Achaete-scute complex-like 1 (Ascl1) orchestrates multiple and in some respect opposing aspects of cortical development such as cellular proliferation and cell cycle exit, as well as neural fate choice (Castro et al., 2011; Guillemot and Hassan, 2017). It is generally believed that oscillating levels of Ascl1 expression promote progenitor proliferation while high and constant levels promote neuronal differentiation (Imayoshi et al., 2013).

In the ventral telencephalon, Ascl1 controls GABAergic neurogenesis by regulating expression of homeobox genes of the distal-less gene family (*Dlx* genes) in progenitors (Casarosa et al., 1999; Poitras et al., 2007). Leveraging its neurogenic activity, we previously demonstrated that expression of Ascl1 in mouse postnatal cortical astrocytes *in vitro* was sufficient to reprogram these into functional neurons endowed with GABAergic neuron properties (Berninger et al., 2007; Heinrich et al., 2010). Likewise, Ascl1 was found to reprogram cultured cells of human origin, including fibroblasts and pericytes, into neurons *in vitro* (Karow et al., 2012; Chanda et al., 2014). Finally, co-expression of Ascl1 and Dlx2 in reactive glia of the adult epileptic hippocampus resulted in the induction of neurons with neurochemical and electrophysiological hallmarks of hippocampal interneurons (Lentini et al., 2021). Beyond its important role in neurogenesis, Ascl1 also plays an important role in gliogenesis. For instance, deletion of Ascl1 was found to cause a decrease in neonatal oligodendrogliogenesis in the dorsal telencephalon, resulting in a relative increase in astrocytes among Ascl1 ablated cells (Nakatani et al., 2013). These studies raise the question whether Ascl1 induces glia-to-neuron conversion *in vivo*, or potentially regulates other aspects of gliogenesis such as proliferation.

Here we addressed this question by injecting Ascl1-encoding retrovirus into the mouse cerebral cortex at postnatal day 5 (P5), i.e., at a time when glial cell populations undergo massive expansion (Psachoulia et al., 2009; Ge et al., 2012). We found that Ascl1 induced only very limited glia-to-neuron reprogramming *in vivo*. In contrast, we observed a drastic increase in proliferative activity in oligodendrocyte progenitor cells (OPCs) but not in astrocytes. These data do not only reveal a rather restricted neuronal reprogramming capacity of Ascl1 when overexpressed in early postnatal astrocytes alone, but also unveils highly divergent responses of distinct glial cell types to this proneural gene.

## Materials and methods

### Cell culture

Postnatal cortical astrocytes were isolated from cortices of C57BL/6J mice between postnatal day 5–7 days (P5–7), which were obtained from the Translational Animal Research Center of the University Medical Center Mainz. P5–P7 astrocytes were cultured as previously described (Heinrich et al., 2011; Sharif et al., 2021). Briefly, after isolation, cells were expanded for 7–10 days in Astromedium: Dulbecco’s Modified Eagles Medium, Nutrient Mixture F12 (DMEM/F12, Gibco, Carlsbad, CA, USA, 21331-020); 10% Fetal Bovine Serum (FBS, Invitrogen, 10270-106); 5% Horse Serum (Thermo Fisher Scientific, Waltham, MA, USA, 16050-130); 1× Penicillin/Streptomycin (Thermo Fisher Scientific, Waltham, MA, USA, 15140122); 1× L-GlutaMAX Supplement (Thermo Fisher Scientific, Waltham, MA, USA, 35050-0380); 1× B27 Supplement (Thermo Fisher Scientific, Waltham, MA, USA, 17504001); and supplemented with 10 ng/μl Epidermal Growth Factor (EGF; Peprotech, Cranbury, NJ, USA, AF-100-15) and 10 ng/μl basic-Fibroblast Growth Factor (FGF-2; Peprotech, Cranbury, NJ, USA, 100-18B). Cells were incubated at 37°C in 5% CO_2_. When cells reached 70–80% confluency, cells were detached with 0.05% Trypsin EDTA (Life Technologies, Carlsbad, CA, USA, 15400054) for 5 min at 37°C. Cells were subsequently seeded onto poly-D-lysine hydrobromide-coated (PDL; Sigma, Merck, Germany, P0899) glass coverslips (12 mm, Menzel-Gläser, Thermo Fisher Scientific, Waltham, MA, USA, 631-0713) in 24-well plates at a density of 50,000–80,000 cells/well in 500 μl Astromedium supplemented with 10 ng/μl EGF and 10 ng/μl FGF-2.

### Plasmids and retroviruses

Moloney Murine Leukaemia Virus (MMLV)-based retroviral vectors (Heinrich et al., 2011) were used to express Ascl1 under control of the chicken β-actin promoter with a cytomegalovirus enhancer (pCAG). A green fluorescent protein (GFP) or DsRed reporter was cloned in behind an Internal Ribosome Entry Site (IRES). To generate the pCAG-Ascl1-IRES-DsRed/GFP retroviral constructs, a cassette containing the coding sequences flanked by attL recombination sites was generated through the excision of the coding sequences for Ascl1 from the pCIG2 parental vector (Li et al., 2014) *via Xho*I/*Sal*I double restriction. Isolated fragment was inserted into the pENTRY1A Dual Selection (Thermo Fisher Scientific, Waltham, MA, USA) intermediate vector linearized *via Sal*I. The final retroviral constructs were subsequently obtained via recombination catalyzed by the LR Clonase II (Thermo Fisher Scientific, Waltham, MA, USA, 11791020), which substituted the ccdB cassette in the destination vector pCAG-ccdB-IRES-DsRed or pCAG-ccdB-IRES-GFP with Ascl1 coding sequence. Transduction with MMLV-based retroviral vectors encoding only the fluorescent protein GFP or DsRed behind an IRES under control of pCAG promoter (pCAG-IRES-DsRed/pCAG-IRES-GFP) (Heinrich et al., 2011) was used for control experiments. Viral particles were produced using gpg helper free packaging cells to generate Vesicular Stomatitis Virus Glycoprotein (VSV-G)-pseudotyped retroviral particles (Ory et al., 1996). Viral stocks were titrated by transduction of HEK293 cultures. Viral titers used were in the range of 10^7^ TU/ml.

### Retroviral transduction

After seeding the cells and letting them attach for 4 h in the incubator, cells were transduced with 1 μl retrovirus/well and incubated at 37°C in 8% CO_2_. One day later, treated medium was removed and substituted with 500 μl of B27 Differentiation Medium: DMEM/F12 (Gibco, Carlsbad, CA, USA, 21331-020); 1× Penicillin/Streptomycin (Thermo Fisher Scientific, Waltham, MA, USA, 15140122); 1× L-GlutaMAX Supplement (Thermo Fisher Scientific, Waltham, MA, USA, 35050-0380); 1× B27 Supplement (Thermo Fisher Scientific, Waltham, MA, USA, 17504001). Cells were treated again with 1 μl/well of retrovirus. One day later, the culture volume was brought to 1 ml/well with fresh B27 Differentiation Medium. Cells were kept in culture for a total of 7 days *in vitro* before fixation for immunocytochemical analyses.

### Immunocytochemistry

Cells were fixed with 4% paraformaldehyde (PFA, Sigma, Merck, Germany, P6148) for 10–15 min and washed three times with 1× PBS (Gibco, Carlsbad, CA, USA, 70013-016) before storage at 4°C. Washed cells were first incubated for 1 h at room temperature (RT) with blocking solution [3% bovine serum albumin (BSA; Sigma, Merck, Germany, A7906) and 0.5% Triton X-100 (Sigma, Merck, Germany, X100) in 1× PBS] and then with primary antibodies diluted in blocking solution for 2–3 h at RT. After three washes with 1× PBS, cells were incubated with secondary antibodies for 1 h at RT. Cells were then counterstained with DAPI (Sigma, Merck, Germany, D8417) diluted 1:1,000 in blocking solution, then washed three time in 1× PBS before being mounted with Aqua Polymount (Polysciences, Warrington, PA, USA, 18606-20). The following primary antibodies were used: β-Tubulin III (Mouse IgG2b, 1:1,000; Sigma, Merck, Germany, T8660); Green Fluorescent Protein (GFP, Chicken, 1:300, AvesLab, Davies, CA, USA, GFP-1020); GFAP (rabbit, 1:1,000, Agilent, Santa Clara, CA, USA, Z0334); Red Fluorescent Protein (RFP, rat, 1:400, Proteintech Group Inc., Rosemont, IL, USA, 5F8). Secondary antibodies were diluted 1:1,000 in blocking solution and were conjugated to: A488 anti-chicken (donkey, Jackson Immunoresearch, Ely, UK, 703-545-155); Cy3 anti-mouse (goat, Dianova, Hamburg, Germany, 115-165-166); Cy3 anti-rat (goat, Dianova, Hamburg, Germany, 112-165-167); Cy5 anti-rabbit (goat, Dianova, Hamburg, Germany, 111-175-144).

### Animals and animal procedures

The study was performed in accordance with the guidelines of the German Animal Welfare Act, the European Directive 2010/63/EU for the protection of animals used for scientific purposes and the Animal (Scientific Procedures) Act 1986 and was approved by local authorities (Rhineland-Palatinate State Authority, permit number 23 177 07-G15-1-031; ethical committee of King’s College London and the UK Home Office, permits numbers PD025E9BC and PP8849003). Male and female C57BL/6J pups were purchased with their mother from Janvier Labs (Le Genest-Saint-Isle, France) or Charles River Laboratories (Walden, UK). Male and female transgenic mGFAP-Cre/EGFP mice used in this study for fate-mapping experiments were generated in house. For this, mice in which the expression of Cre recombinase is driven by mouse GFAP promoter [mGFAP-Cre; B6.Cg-Tg(Gfap-cre)77.6 Mvs/2J, JAX024098] (Gregorian et al., 2009) were crossed with an EGFP reporter mouse line [CAG-EGFP; Gt(ROSA)26Sortm1.1(CAG-EGFP)Fsh/Mmjax, JAX032037] (Sousa et al., 2009). Mice were kept in a 12:12 h light-dark cycle in Polycarbonate Type II cages (350 cm^2^). Animals were provided with food and water *ad libitum* and all efforts were made to reduce the number of animals and their suffering. Before the surgery, animals received a subcutaneous injection of Carprofen [Rimadyl^®^ (Zoetis, Parsipanny, NJ, USA), 4 mg/kg of body weight, in 0.9% NaCl (Amresco, VWR International, Radnor, PA, USA)]. Anesthesia was induced by intraperitoneal (i.p.) injection of a solution of 0.5 mg/kg Medetomidin (Pfizer, New York, NY, USA), 5 mg/kg Midazolam (Hameln, Hameln-Germany) and 0.025 mg/kg Fentanyl (Albrecht GmbH, Aulendorf, Germany) in 0.9% NaCl. Viruses were injected in the cerebral cortex using glass capillaries (Hirschmann, Eberstadt, Germany, 9600105) pulled to obtain a 20 μm tip diameter. Briefly, a small incision was made on the skin with a surgical blade and the skull was carefully opened with a needle. Each pup received a volume of 0.5–1 μl of retroviral suspension targeted to the somatosensory and visual cortical areas. After injection, the wound was closed with surgical glue (3 M Vetbond, Thermo Fisher Scientific, Waltham, MA, USA, NC0304169) and anesthesia was terminated by i.p. injection of a solution of 2.5 mg/kg Atipamezol (Pfizer, New York, NY, USA), 0.5 mg/kg Flumazenil (Hameln, Hameln-Germany) and 0.1 mg/Kg Buprenorphin (RB Pharmaceutials, Richmond, VA, USA) in 0.9% NaCl. Pups were left to recover on a warm plate (37°C) before returning them to their mother. Recovery state was checked daily for a week after the surgery.

### Tissue preparation and immunohistochemistry

Animals were lethally anesthetized with a solution of 120 mg/kg Ketamine (Zoetis, Parsipanny, NJ, USA) and 16 mg/kg Xylazine (Bayer, Leverkusen, Germany) (in 0.9% NaCl, i.p.) and transcardiacally perfused with pre-warmed 0.9% NaCl followed by ice-cold 4% paraformaldehyde (PFA, Sigma, Merck, Germany, P6148). The brains were harvested and post-fixed for 2 h to overnight in 4% PFA at 4°C. Then, 40 μm thick coronal sections were prepared using a vibratome (Microm HM650V, Thermo Scientific, Waltham, MA, USA) and stored at −20°C in a cryoprotective solution [20% glucose (Sigma, Merck, Germany, G8270), 40% ethylene glycol (Sigma, Merck, Germany, 324558), and 0.025% sodium azide (Sigma, Merck, Germany, S2202), in 0.5× phosphate buffer 15 mM Na_2_HPO_4_⋅12H_2_O (Merck, Darmstadt, Germany, 10039-32-4); 16 mM NaH_2_PO_4_⋅2H_2_O (Merck, Darmstadt, Germany, 13472-35-0); pH 7.4].

For immunohistochemistry, brain sections were washed three times for 15 min with 1× TBS [50 mM Tris (Thermo Fisher Scientific, Waltham, MA, USA, 15504-020); 150 mM NaCl (Amresco, VWR International, Radnor, PA, USA, 0241); pH7.6] and then incubated for 1.5 h in blocking solution: 5% Donkey Serum (Sigma, Merck, Germany, S30); 0.3% Triton X-100; 1× TBS. Slices were then incubated with primary antibodies diluted in blocking solution for 2–3 h at RT, followed by an overnight incubation at 4°C. After three washing steps with 1× TBS, slices were incubated with secondary antibodies diluted blocking solution for 1 h at RT. Slices were washed twice with 1× TBS, incubated with DAPI dissolved 1:1,000 in 1× TBS for 5 min at RT and washed three times with 1× TBS. For mounting, slices were washed two times with 1× Phosphate Buffer [30 mM Na_2_HPO_4_⋅12H_2_O (Merck, Darmstadt, Germany, 10039-32-4); 33 mM NaH_2_PO_4_⋅2H_2_O (Merck, Darmstadt, Germany, 13472-35-0); pH 7.4] and were dried on Superfrost (Thermo Fisher Scientific, Waltham, MA, USA) microscope slides. Sections were further dehydrated with toluene and covered with cover-glasses mounted with DPX mountant for histology (Sigma, Merck, Germany, 06522) or directly mounted with Prolong™Gold (Thermo Fisher Scientific, Waltham, MA, USA, P36930). The following primary antibodies were used: Achaete-scute complex-like 1 (Ascl1, mouse IgG1, 1:400, BD Pharmingen, Franklin Lakes, NJ, USA, 556604); Doublecortin (DCX, goat, 1:250, Santa Cruz Biotechnology, Dallas, TX, USA, sc-8066); Green Fluorescent Protein (GFP, chicken, 1:1,000, AvesLab, Davies, CA, USA, GFP-1020); Glial Fibrillary Acidic Protein (GFAP, rabbit, 1:300, Agilent, Santa Clara, CA, USA, Z0334); Ionized calcium-binding adapter molecule 1 (Iba1, rabbit, 1:800, Agilent, Santa Clara, CA, USA, 16A11); mCherry (chicken, 1:300, EnCor Biotechnology, Gainsville, FL, USA, CPCA-mCherry); Red Fluorescent Protein (RFP, rabbit, 1:500, Biomol, Hamburg, Germany, 600401379S); and SRY-Box 10 (Sox10, goat, 1:100, Santa Cruz Biotechnology, Dallas, TX, USA, sc-17342). Secondary antibodies were made in donkey and conjugated with: A488 (anti-chicken, 1:200, Jackson Immunoresearch, Ely, UK, 703-545-155); A488 (anti-rabbit, 1:200, Thermo Fisher Scientific, Waltham, MA, USA, A21206); A647 (anti-rabbit, 1:500, Thermo Fisher Scientific, Waltham, MA, USA, A31573); A488 (anti-mouse, 1:200, Thermo Fisher Scientific, Waltham, MA, USA, A21202); A647 (anti-mouse, 1:500, Thermo Fisher Scientific, Waltham, MA, USA, A31571); Cy3 (anti-chicken, 1:500, Dianova, Hamburg, Germany, 703-165-155); Cy3 (anti-goat, 1:500, Dianova, Hamburg, Germany, 705-165-147); Cy3 (anti-mouse, 1:500, Thermo Fisher Scientific, Waltham, MA, USA, A10037); Cy3 (anti-rabbit, 1:500, Dianova, Hamburg, Germany, 711-165-152); and Cy5 (anti-goat, 1:500, Dianova, Hamburg, Germany, 705-175-147).

### 5-Ethynyl-2′-deoxyuridine incorporation assay

Animals received a single injection of 50 mg/kg (in 0.9% NaCl and 0.25% DMSO, i.p.) 5-Ethynyl-2′-deoxyuridine (EdU, Sigma, Merck, Germany, 900584) 3 h prior to perfusion at 12 days post-injection. The immunohistochemistry protocol was modified as follows for combinatorial detection of EdU: brain sections were washed three times with TBS and then incubated for 2 h in blocking solution [2.5% Donkey Serum, 2.5% Goat Serum (when no staining for Sox10; Sigma, Merck, Germany, G9023), 0.3% Triton X100 in TBS] at RT. Sections were incubated with primary antibodies diluted in blocking solution for 2 h at RT followed by an overnight incubation at 4°C (when no staining for Sox10), or 72 h at 4°C (when staining for Sox10). After three washes with TBS, the sections were incubated for 30 min at RT with the Click-iT EdU Imaging Kit Reaction Cocktail (for 500 μl: 430 μl of 1× Click iT EdU Reaction buffer, 20 μl of CuSO_4_, 1.2 μl Alexa Fluor Azide 647, 50 μl Click iT EdU buffer additive; Thermo Fisher Scientific, Waltham, MA, USA, C10340). Finally, the sections were washed three times with TBS, incubated for 5 min at RT with DAPI (5 μM in PBS) and washed again three times with TBS before being mounted using Mowiol (Cat# 17951-500, Polysciences, Warrington, PA, USA) supplemented with DABCO (Cat# 15154-500, Polysciences, Warrington, PA, USA). The following primary antibodies were used: Red Fluorescent Protein (RFP, rabbit, 1:500, Biomol, Hamburg, Germany, 600401379S); SRY-Box 10 (Sox10, goat, 1:300, R&D Systems, Minneapolins, MN, USA, AF2864). The following secondary antibodies were used: goat Anti-Rabbit A568 (1:1,000; Thermo Fisher, Waltham, MA, USA, A-11011), Donkey anti-Rabbit Cy3 (1:1,1000, Jackson Immunoresearch, Ely, UK, 711-165-152) and Donkey anti-Goat A647 (1:1,000; Thermo Fisher Scientific, Waltham, MA, USA, AB2535864) (A647, 1:1,000).

### Imaging and data analysis

Confocal images were acquired using a TCS SP5 (Leica Microsystems, Wetzlar, Germany) confocal microscope (Institute of Molecular Biology, Mainz, Germany) equipped with four PMTs, four lasers (405 Diode, Argon, HeNe 543, HeNe 633) and a fast-resonant scanner using a 20× dry objective (NA 0.7) or a 40× oil objective (NA 1.3), or with a Zeiss LSM 800 confocal microscope (Carl Zeiss Microscopy, Jena, Germany) equipped with four solid-state lasers (405, 488, 561, and 633 nm) at a 20× objective (NA 0.8) (Centre for Developmental Neurobiology, King’s College London). Alternatively, images were acquired with a Zeiss Axio Imager.M2 fluorescent microscope equipped with an ApoTome (Carl Zeiss Microscopy, Jena, Germany) at a 20× dry objective (NA 0.7) or a 63× oil objective (NA 1.25). For imaging of brain sections, serial Z-stacks spaced at 0.3–2.13 μm distance were acquired to image the whole thickness of the section.

For *in vitro* experiments, biological replicates (n) were obtained from independent cultures prepared from different animals. For each *n*, the value corresponds to the mean value of two technical replicates (i.e., two coverslips). Cell quantifications were performed on 4 × 4 tile scans (individual tile size: 624.70 μm × 501.22 μm). For *in vivo* experiments, *n* corresponds to the number of animals. Quantifications were performed on equally spaced sections (240 or 480 μm) covering the whole area with transduced cells. For fate-mapping experiments in mGFAP-Cre/EGFP mice, cells were quantified in 3–5 sections per animal.

For images used for illustration, the color balance of each channel was uniformly adjusted in Photoshop (Adobe, Mountain View, CA, USA). If necessary, Lookup Tables were changed to maintain uniformity of color coding within figures. When appropriate, a median filter (despeckle) was applied in Fiji (Fiji.sc) to pictures presenting salt-and-pepper noise, and noise was filtered via removal of outlier pixels.

### Statistical analysis

The number of independent experiments (*n*) and number of cells analyzed are reported in the main text or figure legends. Data are represented as means ± SD. Statistical analysis was performed in SPSS Statistics 23 V5 (IBM, Armonk, NY, USA). Normality of distribution was assessed using Shapiro–Wilk test and the significance of the differences between control and Ascl1 groups was analyzed by *t*-test for independent samples or Mann–Whitney *U* test (for normally and non-normally distributed data, respectively). *P*-values are indicated in the figures. Graphs were prepared in GraphPad Prism 5 (GraphPad, San Diego, CA, USA).

## Results

### Achaete-scute complex-like 1 converts postnatal cortical glia into neurons with very low efficiency *in vivo*

**FIGURE 1:**
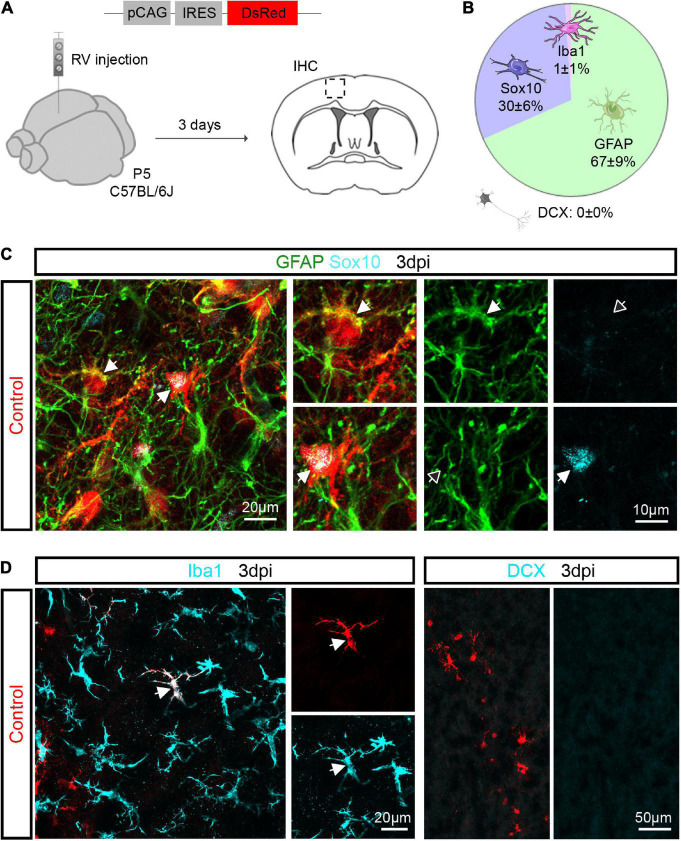
Retroviruses injected in the postnatal cerebral cortex selectively transduce glial cells. **(A)** Experimental scheme. A control retrovirus pCAG-IRES-DsRed was injected in the cerebral cortex of P5 mice and immunohistochemical analysis was performed 3 days later. **(B)** Pie chart showing the relative number of oligodendroglial (Sox10-positive), astroglial (GFAP-positive), microglial (Iba1-positive) and neuronal (DCX-positive) cells among transduced cells. **(C)** Confocal images depicting control-transduced cells (in red, arrows) co-expressing GFAP (in green, upper insets) or Sox10 (in cyan, lower insets). **(D)** Confocal images depicting control-transduced cell (in red) co-expressing Iba1 (in cyan) (left panel). No control-transduced cells expressing DCX were found (in cyan) (right panel). Empty arrows indicate marker-negative cells. IHC, immunohistochemistry; RV, retrovirus; dpi, days post injection.

Our earlier work showed that Ascl1 can reprogram cultured postnatal astroglia into neurons (Berninger et al., 2007; Heinrich et al., 2010; Gascon et al., 2016). Here, we investigated whether Ascl1 can reprogram proliferative cortical glia toward a neuronal fate *in vivo*. Cortical glia massively expands during the first postnatal week by local proliferation and can be targeted by retroviral vectors (Ge et al., 2012; Clavreul et al., 2019). Thus, retroviruses may serve as suitable vectors to test the hypothesis that forced expression of Ascl1 can induce neuronal reprogramming. To validate the approach, we first injected a control virus encoding only a reporter gene (pCAG-IRES-DsRed) into the mouse cerebral cortex at P5 and assessed the identity of the transduced cells by immunohistochemical analysis at 3 days post injection (3 dpi) (Figure 1A). We found that virtually all transduced cells were immunopositive for glial markers (Figure 1B). The majority of transduced cells were immunoreactive for the astroglial marker GFAP (67.0 ± 8.9%, 753 transduced cells analyzed, *n* = 3 mice; Figures 1B,C), and the remaining were oligodendroglial cells immunoreactive for Sox10 (29.6 ± 6.1%, 753 transduced cells analyzed, *n* = 3 mice; Figures 1B,C). Rarely, we found transduced cells immunoreactive for the microglial marker Iba1 (0.9 ± 1.0%, 578 transduced cells analyzed, *n* = 3 mice; Figures 1B,D). Importantly, none of the control-transduced cells expressed the immature neuronal marker DCX (0.0 ± 0.0%, 578 transduced cells analyzed, *n* = 3 mice; Figures 1B,D). These results indicate that retroviruses injected in the P5 mouse cerebral cortex *in vivo* specifically transduce astroglial and oligodendroglial lineage cells.

**FIGURE 2:**
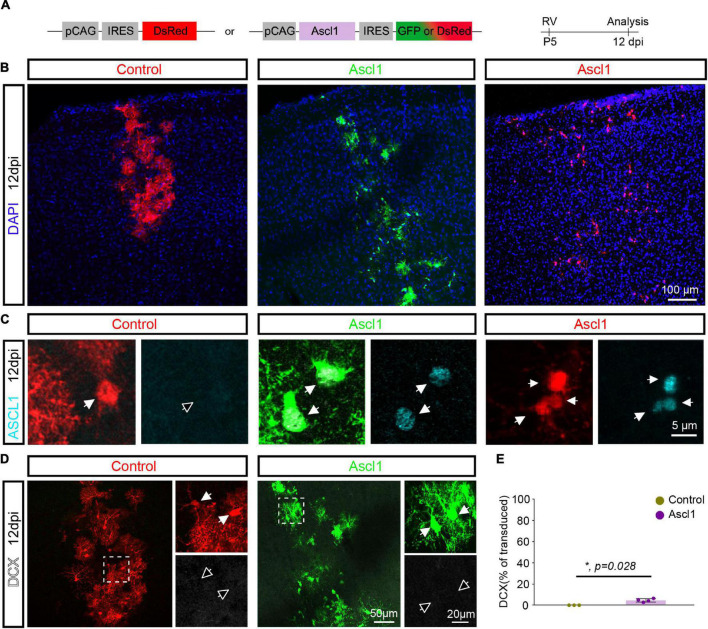
Achaete-scute complex-like 1 (Ascl1) converts postnatal glia into neurons with low efficiency *in vivo*. **(A)** Experimental scheme. A control (pCAG-IRES-DsRed) or Ascl1-encoding (pCAG-Ascl1-GFP or pCAG-Ascl1-DsRed) retrovirus was injected in the cerebral cortex of P5 mice and immunohistochemical analysis was performed 12 days later. **(B)** Low-magnification confocal images depicting transduced cells at cortical site of injection. **(C)** Immunohistochemistry confirmed the lack of expression of Ascl1 in control-transduced postnatal cortical glia and efficient Ascl1 induction (in cyan) by Ascl1-encoding retroviruses. **(D)** Confocal images depicting the maintenance of a glial morphology and lack of DCX induction (in white) in control or Ascl1-transduced cells. Empty arrows indicate marker-negative cells. **(E)** Quantification of the percentage of transduced cells expressing DCX at 12dpi indicates that Ascl1 induces neurogenesis from postnatal cortical glia with low efficiency. Mean ± SD, Mann–Whitney *U* test. RV, retrovirus; dpi, days post injection.

To examine the consequences of forced expression of Ascl1, we next injected control (pCAG-DsRed) or Ascl1-encoding (pCAG-Ascl1-DsRed or pCAG-Ascl1-GFP) retrovirus and investigated whether the proneural factor could reprogram P5 proliferative glia into neurons using immunohistochemistry (Figure 2A). Analysis was performed at 12 dpi, based on previous evidence of retrovirus-mediated glia-to-neuron reprogramming within 7–14 days *in vivo* (Heinrich et al., 2014; Gascon et al., 2016; Herrero-Navarro et al., 2021). Many reporter-positive cells were found at the site of retrovirus injection (Figure 2B), and Ascl1 was effectively expressed in cells transduced with Ascl1, while it was absent from control-transduced cells (Figure 2C). Control-transduced cells lacked DCX expression (0.0 ± 0.0%, 2,157 transduced cells analyzed, *n* = 3 mice; Figures 2D,E), confirming that the control vector did not induce a cell fate switch. Surprisingly, Ascl1-transduced cells also largely remained immunonegative for DCX (Figures 2D,E), with only a small, albeit statistically significant number of transduced cells exhibiting an immature neuron-like morphology and expressing DCX (Ascl1: 4.6 ± 1.6%, 720 transduced cells analyzed, *n* = 3 mice) (Figures 2D,E). To confirm the biological activity of our Ascl1-encoding retrovirus, we transduced cultures of cortical astrocytes with control (pCAG-DsRed or pCAG-GFP) or Ascl1-encoding (pCAG-Ascl1-DsRed) retrovirus (Supplementary Figure 1A). Following transduction with control virus, virtually no β-Tubulin III-immunoreactive cells were found (0.1 ± 0.2%, 1,398 transduced cells analyzed, *n* = 3 biological replicates; Supplementary Figures 1B,C). In contrast, astrocytes transduced with Ascl1-encoding retrovirus acquired neuron-like morphology and expressed β-Tubulin III (27.3 ± 3.8%, 3,061 transduced cells analyzed, *n* = 4 biological replicates; Supplementary Figures 1B,C). Together, our results indicate that despite the neurogenic potential of Ascl1 *in vitro*, *in vivo* reprogramming by Ascl1 by and large fails.

### Achaete-scute complex-like 1 expression in postnatal cortical glia increases the relative number of cells expressing oligodendroglial markers

**FIGURE 3:**
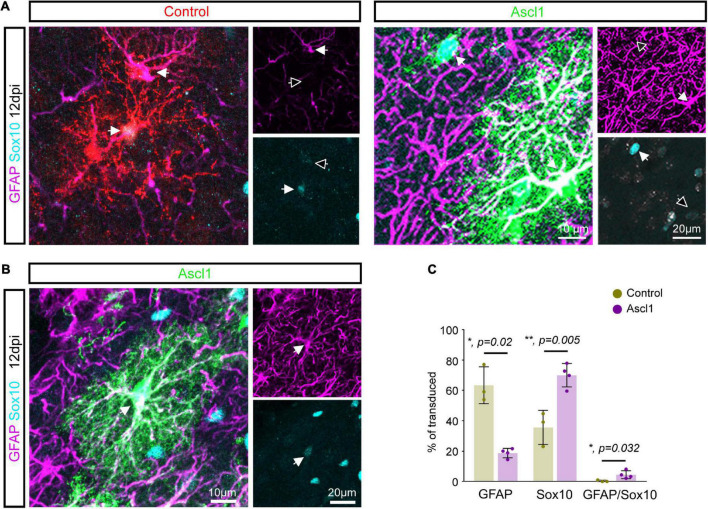
Achaete-scute complex-like 1 (Ascl1) induces an increase in the number of Sox10 expressing cells. **(A)** Confocal images depicting control and Ascl1-transduced cells expressing either GFAP (in magenta) or Sox10 (in cyan). **(B)** Confocal images depicting Ascl1-transduced cells co-expressing GFAP (in magenta) and Sox10 (in cyan). **(C)** Quantification of the percentage of transduced cells expressing GFAP, Sox10 or both markers at 12 dpi indicates a concomitant reduction in the relative number of cells expressing an astroglial marker and increase in the relative number of cells expressing an oligodendroglial marker upon transduction with Ascl1. Empty arrows indicate marker-negative cells. Mean ± SD, *t*-test for independent samples (for GFAP and Sox10) and Mann–Whitney *U* test (for GFAP/Sox10).

Given that only a few Ascl1-transduced cells were converted into neurons, we examined whether the remainder of the transduced cells nevertheless had responded to Ascl1 with downregulation of glial markers. We therefore analyzed the expression of the pan-oligodendroglial marker Sox10 and the astroglial marker GFAP in Ascl1-transduced cells (Figures 3A–C). Consistent with our analysis at 3 dpi (Figure 1), control-transduced cells at 12 dpi were glial cells, with two thirds of the cells expressing the astroglial marker GFAP (63.3 ± 12.1%, 1,885 transduced cells analyzed, *n* = 3 mice) while the other third expressed Sox10 (35.6 ± 11.3%, 1,885 transduced cells analyzed, *n* = 3 mice; Figures 3A,C). As expected, the expression of GFAP and Sox10 was mutually exclusive in control-transduced cells (0.3 ± 0.6% of GFAP/Sox10-positive cells, 1,885 transduced cells analyzed, *n* = 3 mice; Figures 3B,C and Supplementary Movie 1). Following transduction with Ascl1-encoding virus, we observed a marked alteration in the relative expression of glial markers. Strikingly, only one fifth of transduced cells exclusively expressed GFAP (Ascl1, 18.7 ± 3.1%, 848 transduced cells analyzed, *n* = 4 mice), a three folds reduction compared to control transductions. Interestingly, the reduction in GFAP expression was concomitant with a two folds increase in the relative number of Sox10-only expressing cells (70.0 ± 7.7%, 848 transduced cells analyzed, *n* = 4 mice; Figure 3C). Moreover, a modest but significant increase in the relative number of cells co-expressing Sox10 and GFAP was observed in Ascl1-transduced cells (Figures 3B,C and Supplementary Movie 2). The detection of GFAP/Sox10-immunopositive cells following transduction with Ascl1-encoding virus (4.5 ± 2.6%, 848 transduced cells analyzed, *n* = 4 mice, Figures 3B,C and Supplementary Movie 2) may capture cells in a “mixed” glial state. Together, these results indicate that although largely failing to redirect proliferative glial cells toward neurogenesis, these cells appear to be responsive to Ascl1 overexpression. Changes in the relative numbers of Sox10- vs. GFAP-positive cells could be accounted for either by: (i) conversion of the astroglial lineage toward the oligodendroglial lineage; (ii) glial cell type-specific changes in the rates of proliferation (and/or death) of cells of the oligodendroglial or astroglial lineage, respectively.

### Genetic fate mapping argues against massive astrocyte-to-oligodendrocyte progenitor cell conversion following Achaete-scute complex-like 1 overexpression

**FIGURE 4:**
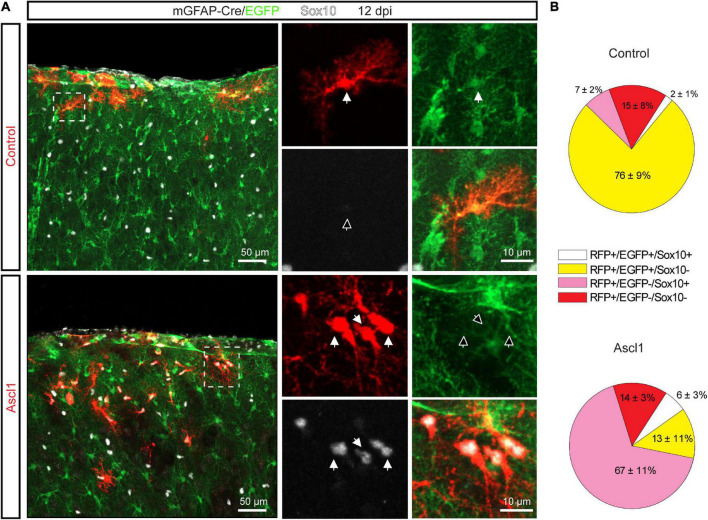
The increase in the relative proportion of Sox10-expressing cells is not due to Achaete-scute complex-like 1 (Ascl1) mediated conversion of astrocytes into OPCs. **(A)** Confocal images depicting control- and Ascl1-transduced cells (in red) in mGFAP-Cre/EGFP mice at 12 dpi. Empty arrows indicate marker-negative cells. **(B)** Pie charts showing the relative number of transduced cells co-expressing EGFP and/or Sox10 at 12 days following transduction with control (upper pie chart) or Ascl1 (lower pie chart) retrovirus. Mean ± SD. Dpi, days post injection.

Intrigued by this finding, we performed genetic fate mapping experiments to follow the fate of transduced astroglia employing mGFAP-Cre/EGFP mice, in which permanent green-fluorescent labeling was specifically achieved in cells with an active mGFAP promoter (i.e., astrocytes) by Cre-mediated removal of the loxP-flanked STOP cassette upstream of EGFP. We injected control (pCAG-DsRed) or Ascl1-encoding (pCAG-Ascl1-DsRed) retrovirus in the cortex of P5 mGFAP-Cre/EGFP mice and quantified at 12 dpi the relative proportion of cells expressing EGFP and/or Sox10 among RFP-positive transduced cells (Figure 4). In line with our immunohistochemical analysis (Figure 3), most cells transduced with control retrovirus were EGFP-positive (585 transduced cells analyzed, *n* = 3 mice, Figure 4B, upper pie chart, yellow and white pie chart sectors), indicative of astroglial identity. While we previously had not observed cells co-expressing GFAP and Sox10 in control transductions (Figure 3), we recorded here a minor proportion of control transduced cells co-expressing EGFP and Sox10. The remainder of the transduced cells were not fate-mapped (EGFP-negative) and, as expected, partly composed of Sox10-positive oligodendroglial cells (Figure 4B, upper pie chart, pink pie chart sector). In accordance with our immunohistochemical analysis (Figure 3), we noted a very different relative distribution following transduction with Ascl1 (878 transduced cells analyzed, *n* = 3 mice, Figure 4B, lower pie chart). Similar to our earlier analysis, we found that a larger proportion of Ascl1-transduced cells were Sox10-positive (Figure 4B, lower pie chart, white and pink pie chart sector), as compared with control. Sox10-positive cells were also detected among the EGFP-positive cells (Figure 4B, lower pie chart, white pie chart sector), in line with the observation that some Ascl1 transduced cells expressed both GFAP and Sox10. Most importantly, however, our analysis showed that the vast majority of Sox10-positive cells were still EGFP-negative (Figures 4A,B, lower pie chart, pink pie chart sector). Taken together, these data indicate that the observed increase in the relative proportion of Sox10-expressing oligodendroglia among Ascl1-retrovirus transduced cells is by and large not attributable to direct lineage conversion of astrocytes into oligodendroglial cells.

### Achaete-scute complex-like 1 induces proliferation in Sox10-positive oligodendrocyte progenitor cells

**FIGURE 5:**
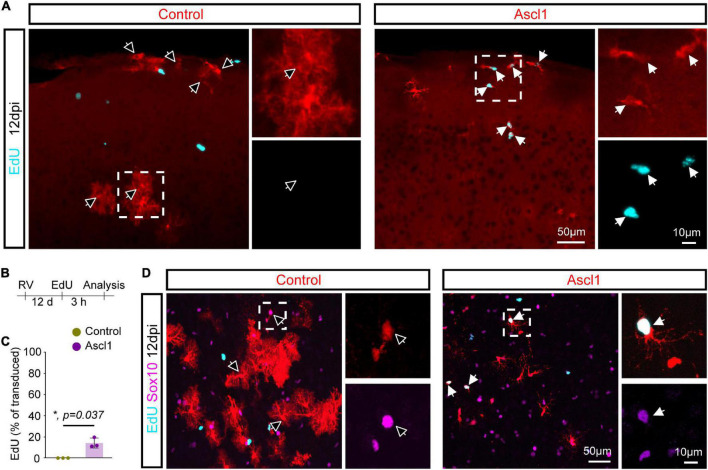
Achaete-scute complex-like 1 (Ascl1)-overexpresion increases proliferative activity of Sox10-possitive cells. **(A)** Confocal images depicting incorporation of EdU by transduced cells. **(B)** Experimental scheme. EdU was injected to the mice 3 h prior to sacrifice 12 days after retrovirus injection. **(C)** Quantification of the percentage of transduced cells that have incorporated EdU indicates that, in contrast to control cells, some Ascl1-transduced cells are maintained in a proliferative state. Mean ± SD, Mann–Whitney *U* test. **(D)** Confocal images depicting Sox10 expression in EdU-positive transduced cells. Empty arrows indicate EdU-negative cells. RV, retrovirus; dpi, days post injection.

To examine the alternative possibility that Ascl1 promoted a higher proliferation rate in OPCs vs. astrocytes, we pulse-labeled proliferating cells by systemic injection of the thymidine analogue EdU 3 h prior to sacrifice at 12 dpi (Figures 5A,B). Expansion of cortical glia rapidly declines after the first two postnatal weeks (Psachoulia et al., 2009; Ge et al., 2012; Clavreul et al., 2019). Accordingly, we found that none of the control-transduced cells had incorporated EdU (0.0 ± 0.0%, 218 transduced cells analyzed, *n* = 3 mice; Figures 5A,C). Strikingly, the 3 h EdU pulse resulted in labeling of a significant proportion of Ascl1-transduced cells (14.1 ± 4.7%, 177 transduced cells analyzed, *n* = 3 mice; Figures 5A,C). Analysis of the identity of the EdU-positive Ascl1-transduced cells showed that virtually all expressed Sox10 (96.7 ± 5.8%, 56 EdU-positive Ascl1-transduced cells analyzed, *n* = 3 mice; Figure 5D), indicative of a specific effect on the proliferative status of the oligodendroglial lineage. Together, our data indicates that Ascl1 promotes cell cycle activity selectively in cells of the oligodendroglial and not the astroglial lineage, thereby accounting for the change in the relative numbers of these two lineages following Ascl1 overexpression *in vivo*.

## Discussion

In the present study, we assessed the effect of Ascl1 overexpression in glia during their proliferative expansion phase in the early postnatal cerebral cortex. In contrast to earlier findings *in vitro* (Berninger et al., 2007; Heinrich et al., 2010; Gascon et al., 2016)—which we confirmed herein—we found that glia-to-neuron conversion by Ascl1 was very inefficient in the early postnatal cortex *in vivo*. However, Ascl1 overexpression shifted the number of Sox10-positive OPCs vs. GFAP-positive astrocytes among transduced cells. While a minor contribution of astrocyte-to-OPC conversion might have occurred, this effect could be attributed by and large to increased cell cycle activity in OPCs as shown by genetic fate mapping and EdU incorporation experiments. Overall, these data indicate that Ascl1 differentially affects cell cycle activity in distinct glial cell types, highlighting the importance of cellular context for the consequences of Ascl1 overexpression.

The low rate of glia-to-neuron conversion triggered by Ascl1 *in vivo* is in agreement with previous studies reporting inefficient neuronal reprogramming following retrovirus- or lentivirus-mediated expression of Ascl1 alone in reactive glia in the adult lesioned cortex (Heinrich et al., 2014), adult striatum (Niu et al., 2015), and adult lesioned spinal cord (Su et al., 2014). In contrast to these studies, another study reported very efficient reprogramming of astrocytes into mature neurons following adeno-associated virus (AAV)-mediated expression of Ascl1 in the dorsal midbrain, striatum and somatosensory cortex (Liu et al., 2015). However, misidentification of endogenous neurons as glia-derived neurons was recently reported following AAV-mediated expression of Neurod1, possibly due to transgene sequence-specific effects acting *in cis* (Wang et al., 2021). Thus, one possible explanation for the apparent discrepancy in reprogramming efficiency *in vivo* is that, similarly to Neurod1, AAV-mediated expression of Ascl1 resulted in labeling of endogenous neurons. Future studies combining AAV-mediated expression of reprogramming factors such as Ascl1 with genetic lineage tracing are required to clarify the origin of seemingly induced neurons (Wang et al., 2021; Leaman et al., 2022).

The apparent difference in reprogramming potency of Ascl1 *in vitro* and *in vivo* could be attributed to various factors: (i) Enhanced cellular plasticity of cultured astrocytes as compared to astrocytes *in vivo* despite both being of similar age and in a similar proliferative state. The protocol employed here to culture and reprogram astrocytes may enhance their competence to undergo cell fate conversion. Indeed, a previous study showed that allowing astrocytes to mature *in vitro* for few days prior to proneural factor activation resulted in a drastic decrease in reprogramming rate, an effect that could be attributed to activation of the REST/coREST repressor complex and accompanying epigenetic maturation (Masserdotti et al., 2015). *In vivo*, REST/coREST complex activity may be already higher, thereby safeguarding astrocyte identity against Ascl1-induced neurogenic reprogramming. (ii) Another important difference is the obviously more complex local environment *in vivo*. Nearly nothing is known about the influence that other cell types exert on cells that undergo reprogramming. However, *in vitro* studies have shown that human pericytes undergoing reprogramming by Ascl1 and Sox2 pass through a neural stem cell-like stage during which they become responsive to several intercellular signaling pathways including Notch signaling (Karow et al., 2018). Thus, it is conceivable that signaling molecules as well as extracellular matrix components secreted by cells within the local environment could impinge on early and perhaps more vulnerable reprogramming stages, thereby curtailing progression toward neurogenesis. The overall very low conversion efficiency suggests that glial cells possess effective safeguarding mechanisms that protect them against acquiring a neurogenic fate. In fact, these safeguarding mechanisms are effective even when confronted with a powerful transcription factor with pioneer factor activity, such as Ascl1 (Wapinski et al., 2013; Raposo et al., 2015; Park et al., 2017).

While Ascl1 did not induce neurogenic conversion in cells of the astroglial and oligodendroglial lineages, we observed a significant shift in the ratio of virus-transduced astroglial to oligodendroglial cells. This shift can be accounted for primarily by increased proliferation of Sox10-positive OPCs following Ascl1 overexpression, whereas astroglia-to-OPC conversion may have contributed only marginally. The fact that approximately 15% of the Ascl1-expressing OPCs incorporated EdU during a 3 h time window may indicate that this population proliferated homogenously and at a drastically shortened cell cycle length as compared to OPCs under control conditions (Psachoulia et al., 2009). EdU saturation experiments would help to determine the growth fraction of cells actively engaged in cell cycle among all Ascl1-expressing OPCs. Furthermore, it would be of great interest to learn whether Ascl1-overexpressing OPCs eventually exit the cell cycle and differentiate into oligodendrocytes. If so, Ascl1-induced expansion of the OPC pool could be a strategy for regenerating oligodendrocytes in demyelinating diseases. Ascl1-induced OPC cell cycle activity observed here is consistent with earlier findings reporting a physiological role of Ascl1 in regulating OPC proliferation in the adult spinal cord (Kelenis et al., 2018).

Intriguingly, studies in the adult hippocampus have previously reported that similar retroviral expression of Ascl1 in neural stem cells promoted oligodendrogliogenesis instead of GABAergic neurogenesis (Jessberger et al., 2008; Braun et al., 2015). While these data were interpreted as an Ascl1-induced change in cell fate of adult neural stem cells, our data may open the alternative possibility that Ascl1 overexpression enhanced the local proliferation of retrovirus-targeted OPCs, or potentially even a combination of both effects.

Previous work has highlighted the proliferation-promoting role of Ascl1. While critical for neuronal differentiation of ventral telencephalic progenitors, Ascl1 also regulates genes involved in cell cycle regulation, and Ascl1 deletion results in reduced progenitor proliferation (Castro et al., 2011). Moreover, Ascl1 overexpression in embryonic cortical progenitors induces proliferation and the expression of Sox9, a glioblast marker (Li et al., 2014). Likewise, Ascl1 plays a key role in neural stem cell activation in the adult hippocampus (Andersen et al., 2014) while its downregulation promotes return to quiescence (Urban et al., 2016). Furthermore, Ascl1 induction also takes part in the reactivation of a neurogenic program in astrocytes in response to injury or silencing of Notch signaling in the adult striatum or cortex, a process which involves transient proliferation of Ascl1-expressing astrocytes (Magnusson et al., 2014; Nato et al., 2015; Zamboni et al., 2020). Against this context, it is intriguing that we found that the proliferation-promoting effect of Ascl1 is restricted to OPCs when overexpressed at P5, and astrocytes did not enter cell cycle. One speculative possibility is that the differential response of OPCs and astrocytes to Ascl1 depends on temporal expression dynamics. In our experimental conditions, Ascl1 expression is likely to be relatively constant. In contrast, previous studies found that in proliferating neural stem cells, which molecularly are more akin to astrocytes than OPCs, Ascl1 expression undergoes oscillations that are out-of-phase with oscillating effectors downstream of Notch signaling (Imayoshi et al., 2013). Our data provide the first example of proliferation induced by constant and likely high levels of Ascl1 and warrant future studies into the cell type specific sensitivity to Ascl1 expression dynamics.

## Data availability statement

The raw data supporting the conclusions of this article are openly available from the King’s College London research data repository, KORDS, at doi: 10.18742/21552657.

## Ethics statement

The animal study was reviewed and approved by the Rhineland-Palatinate State Authority and the Ethical Committee of King’s College London and the UK Home Office.

## Author contributions

CG, NM, FS, LG, YS, and SP: methodology, investigation, and formal analysis. CS: resources. CG, NM, and SP: writing—original draft. CS, BB, and SP: funding acquisition, conceptualization, visualization, and writing—review and editing. All authors contributed to the article and approved the submitted version.
