# Corrigendum: Enhanced proliferation of oligodendrocyte progenitor cells following retrovirus mediated Achaete-scute complex-like 1 overexpression in the postnatal cerebral cortex *in vivo*

**DOI:** 10.3389/fnins.2023.1198640

**Published:** 2023-04-18

**Authors:** Chiara Galante, Nicolás Marichal, Franciele Franco Scarante, Litsa Maria Ghayad, Youran Shi, Carol Schuurmans, Benedikt Berninger, Sophie Péron

**Affiliations:** ^1^Institute of Physiological Chemistry, University Medical Center Johannes Gutenberg University, Mainz, Germany; ^2^Centre for Developmental Neurobiology, Institute of Psychiatry, Psychology & Neuroscience, King's College London, London, United Kingdom; ^3^Department of Pharmacology, Ribeirão Preto Medical School, University of São Paulo, São Paulo, Brazil; ^4^The Francis Crick Institute, London, United Kingdom; ^5^Biological Sciences Platform, Sunnybrook Research Institute, Toronto, ON, Canada; ^6^Department of Biochemistry, University of Toronto, Toronto, ON, Canada; ^7^Department of Laboratory Medicine and Pathobiology, University of Toronto, Toronto, ON, Canada; ^8^MRC Centre for Neurodevelopmental Disorders, Institute of Psychiatry, Psychology & Neuroscience, King's College London, London, United Kingdom; ^9^Focus Program Translational Neuroscience, Johannes Gutenberg University, Mainz, Germany

**Keywords:** astrocyte, gliogenesis, lineage reprogramming, neurogenesis, proliferation, proneural, Sox10, Ascl1

In the published article, there was an error in the Data Availability Statement. The doi to access to the raw data supporting the conclusions of this article was displayed as “doi: 10.5061/dryad.6gb90”. The correct Data Availability Statement with the correct doi appears below.

The authors state that this error does not change the scientific conclusions of the article in any way. The original article has been updated.

## Data availability statement

The raw data supporting the conclusions of this article are openly available from the King's College London research data repository, KORDS, at doi: 10.18742/21552657.

