# Supplementary material for: Enhanced proliferation of oligodendrocyte progenitor cells following retrovirus mediated Achaete-scute complex-like 1 overexpression in the postnatal cerebral cortex in vivo
Source: Front Neurosci. 2022 Dec 2;16:919462. doi: 10.3389/fnins.2022.919462 (PMC9755855; doi:10.3389/fnins.2022.919462)
Supplement: Supplementary file 1 [file Data_Sheet_1.docx]

Supplementary Material

**1. Methods and Materials**

# Retrieval policy

Studies published from the establishment of the databases to 31 March 2022 were searched in the English databases PubMed, Web of Science, Embase, Cochrane and Chinese databases CNKI, VIP, and Wanfang. The retrieval strategy of “subject words + free words” was adopted, The search terms used are as follows: [“acupuncture” or “electroacupuncture” or “transcutaneous electrical acupoint stimulation (TEAS)” or “auricular acupuncture” or “needle warming moxibustion”] and [“Cancer-related Insomnia” or “tumor” or “cancer” or “neoplasia” or “CRI”] and [“sleep” or “insomnia” or “sleep disorder”]. A preliminary filter using *King of medical literature* identified 1229 related articles.

**Specific retrieval strategy**

**CNKI:**（SU = ‘肿瘤' OR SU = ‘癌' OR SU = ‘癌症' OR SU = ‘恶性肿瘤' OR SU = ‘瘤') AND (SU = ‘针灸' OR SU = ‘针刺' OR SU = ‘针法' OR SU = ‘电针' OR SU = ‘温针灸' OR SU = ‘耳针' OR SU = ‘经皮穴位电刺激' OR SU = ‘头皮针' OR SU = ‘毫针’) AND (SU = ‘失眠' OR SU = ‘睡眠不足' OR SU = ‘睡眠障碍' OR SU = ‘肿瘤相关性失眠') AND FT = ‘随机'

**VIP:**(K=("肿瘤" OR "癌" OR "癌症" OR "恶性肿瘤" OR "瘤")) AND (K=("针灸" OR "针刺" OR "针法" OR "电针" OR "温针灸" OR "耳针" OR "经皮穴位电刺激" OR "头皮针" OR "毫针" OR"耳穴")) AND (K=("失眠" OR "睡眠障碍" OR "睡眠不足" OR "肿瘤相关性失眠")) AND U="随机"

**WANFANG:**(主题:("肿瘤") or 主题:("癌") or 主题:("癌症") or 主题:("恶性肿瘤") or 主题:("瘤")) and (主题:("针灸") or 主题:("针刺") or 主题:("针法") or 主题:("电针") or 主题:("温针灸") or 主题:("耳针") or 主题:("经皮穴位电刺激") or 主题:("头皮针") or 主题:("毫针")) and (主题:("失眠") or 主题:("睡眠障碍") or 主题:("睡眠不足") or 主题:("肿瘤相关性失眠")) and 全部:("随机")

**Pubmed：**
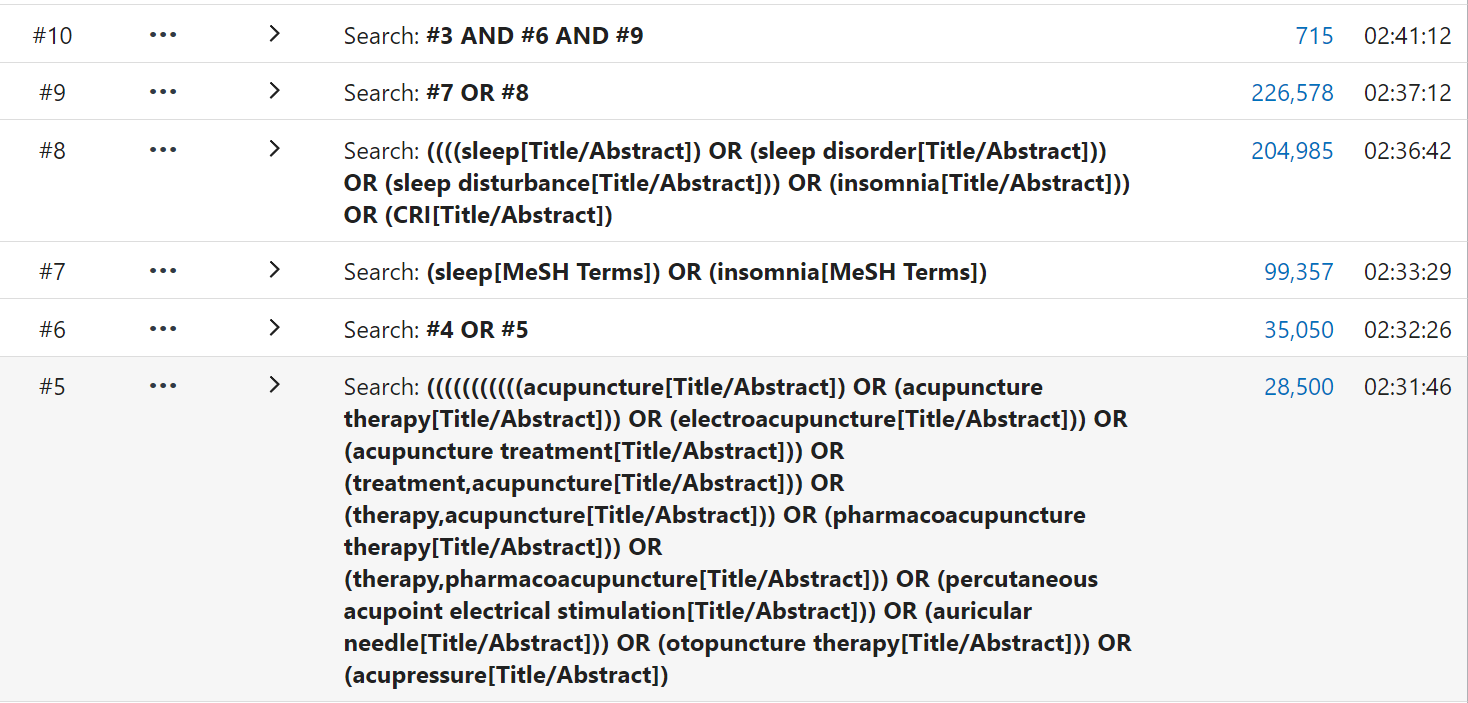


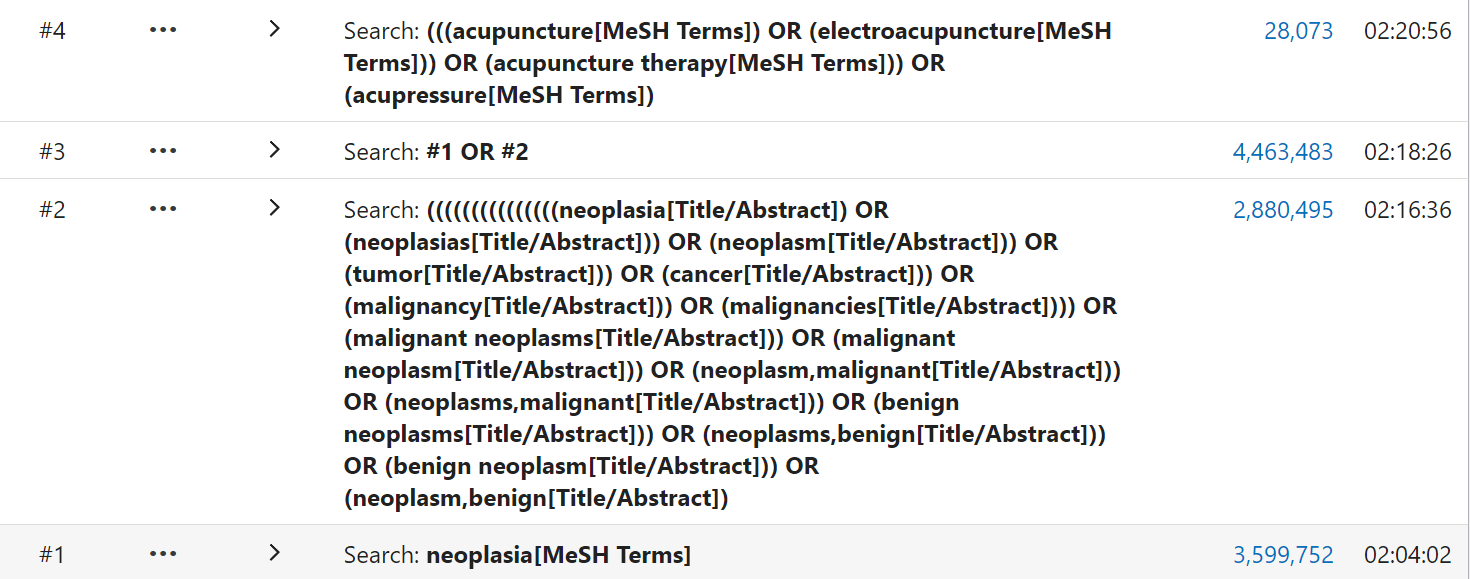


**Web of science：**
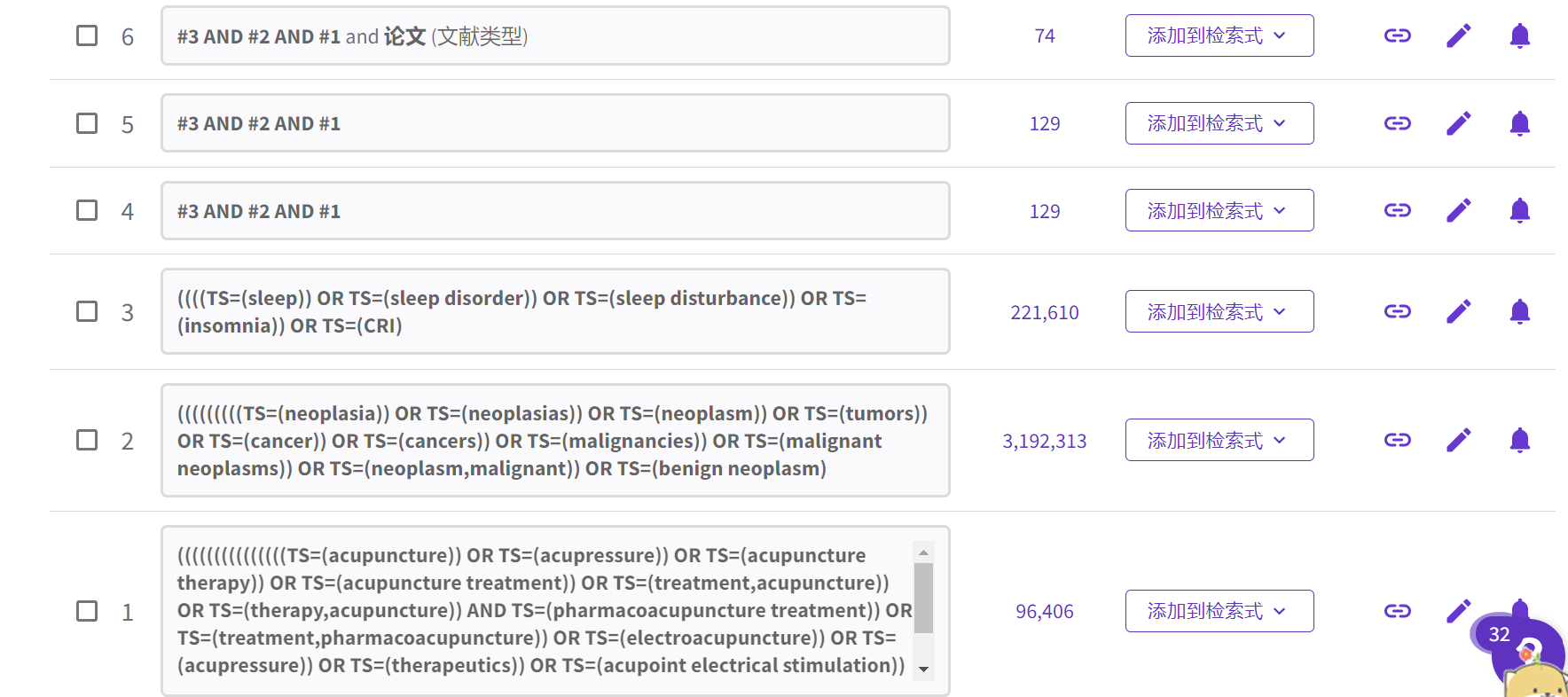


**Cochrane：**
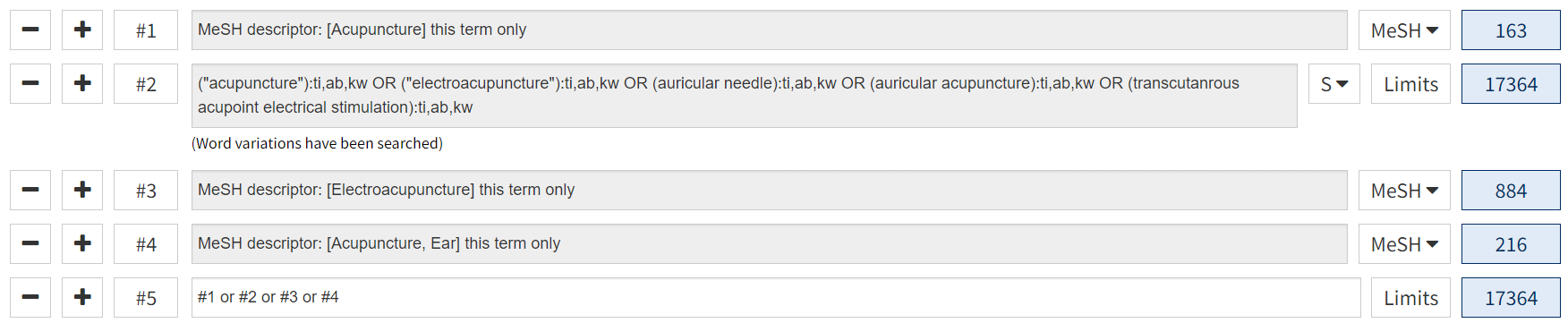


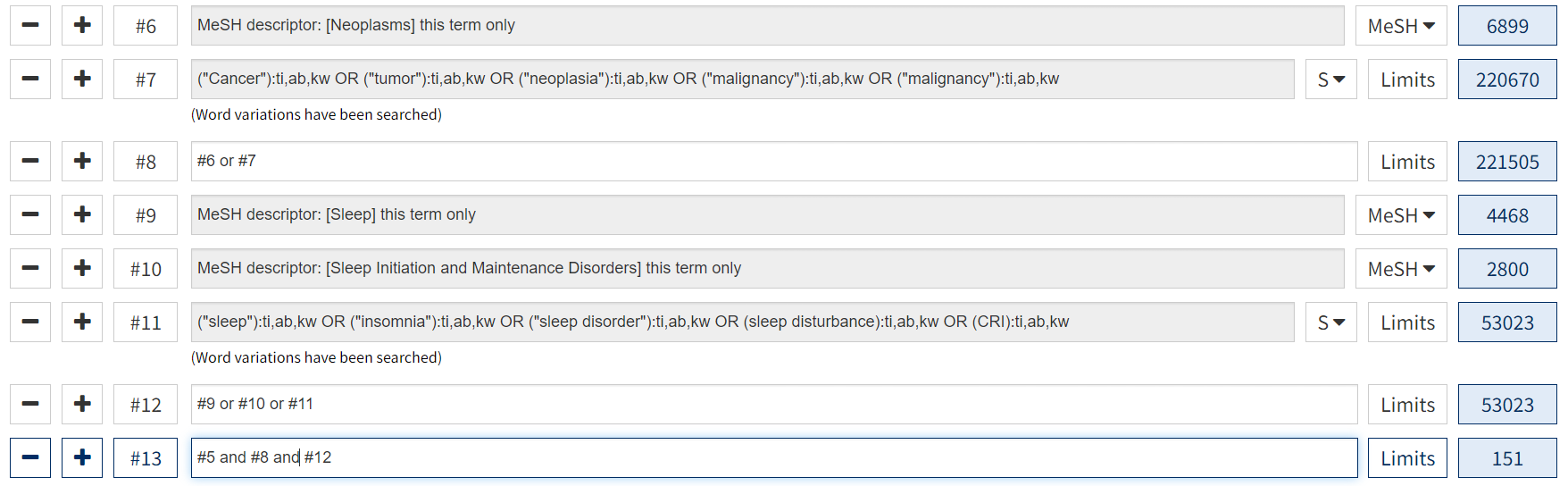


**Embase：**
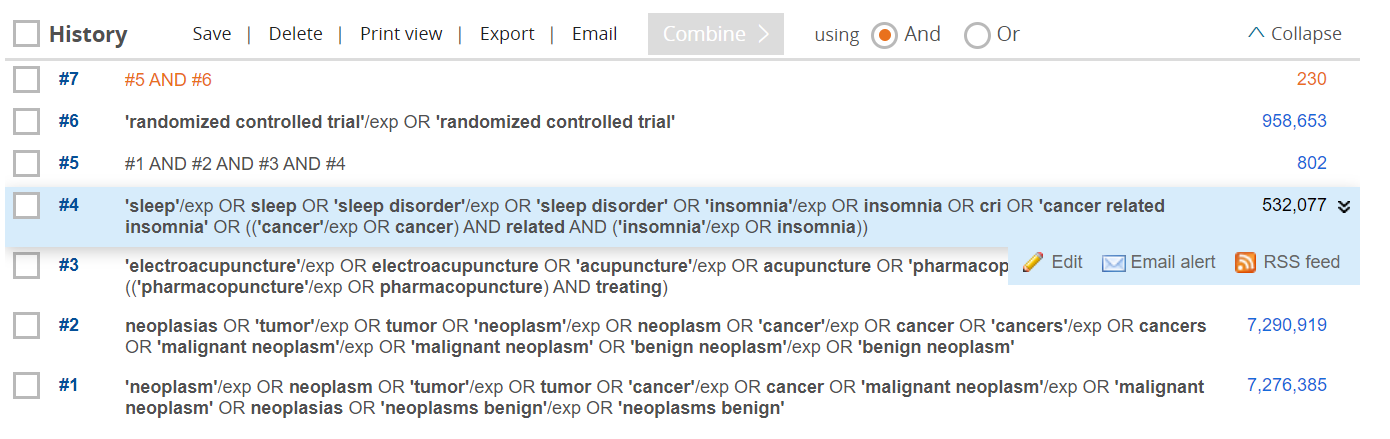


# Supplementary Figures


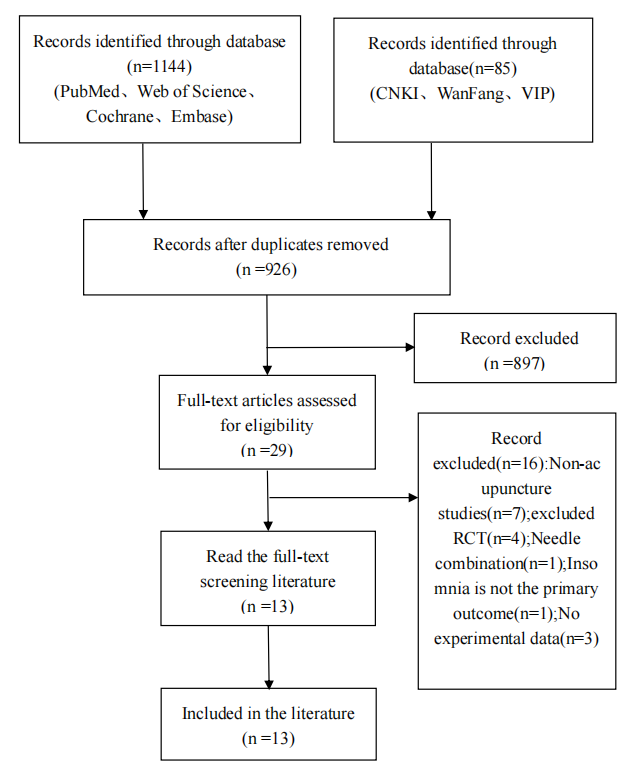


**Figure 1.** PRISMA flow diagram of included articles.

**（A）**
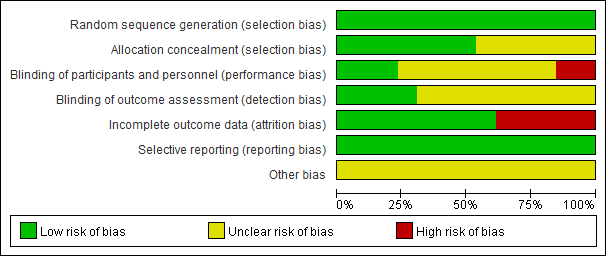


**（B）**
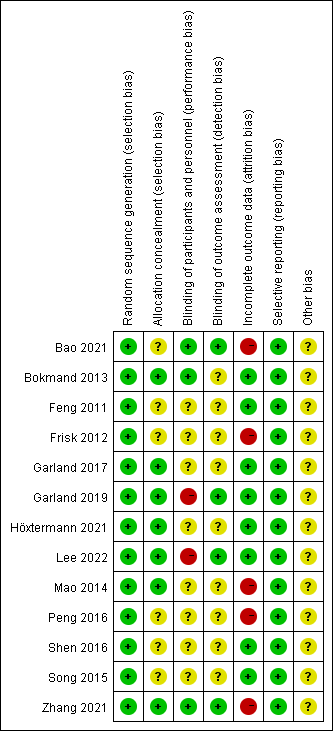


**Figure 2**. Risk of bias of included trials.(**A)**Assessment of risk of bias presented as percentages across all included studies. (**B)**Risk of bias summary for each included study.


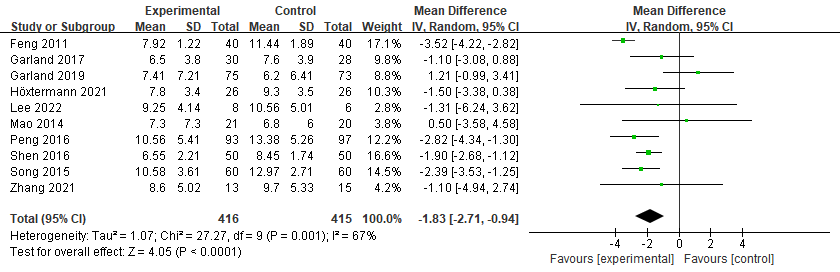


**Figure 3**. Forest plot of PSQI scale scores


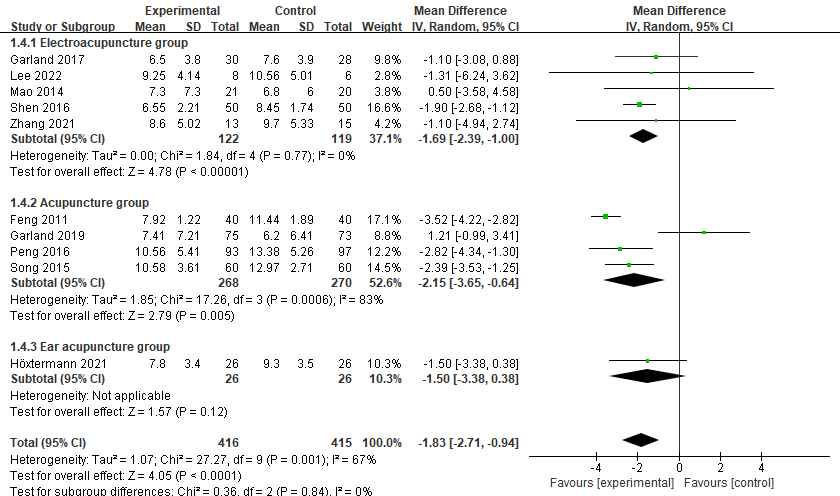


**Figure 4**. Subgroup analysis of PSQI scores

(A)
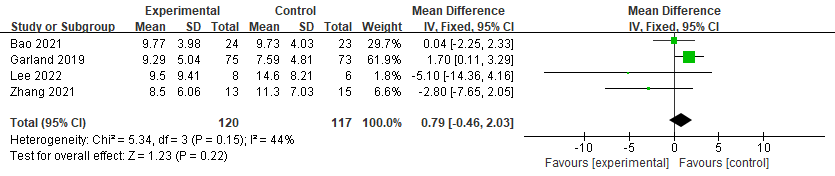


(B)
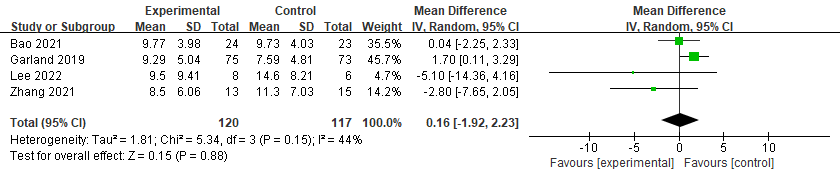


**Figure 5**. Forest plot of ISI scale scores.(A)fixed effect model.(B)the random effect model.


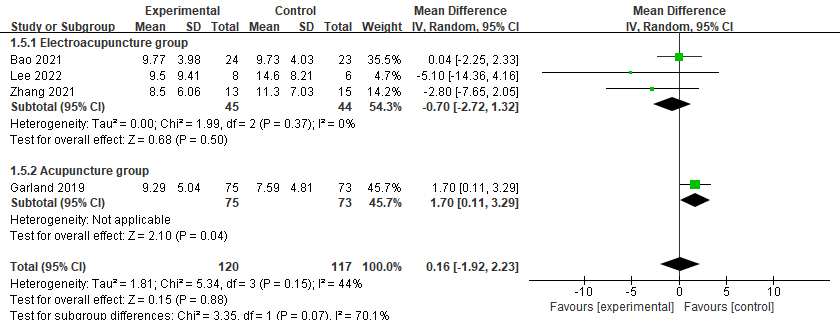


**Figure 6**. Subgroup analysis of ISI scores


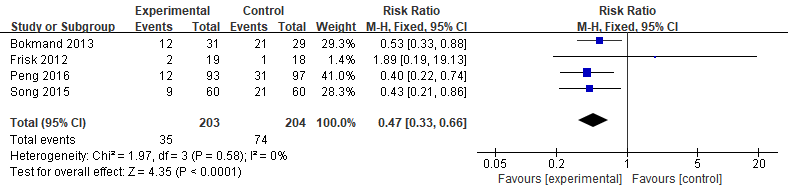


**Figure 7**. Forest plot of efficacy analysis.


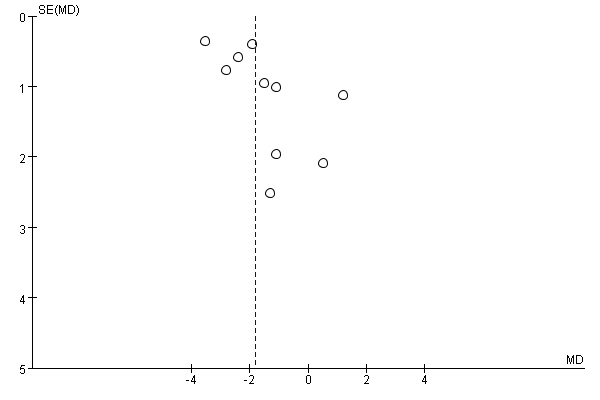


**Figure S1**. Funnel plot of PSQI.
