# Supplementary figures and images for: Enhanced proliferation of oligodendrocyte progenitor cells following retrovirus mediated Achaete-scute complex-like 1 overexpression in the postnatal cerebral cortex in vivo
Source: Front Neurosci. 2022 Dec 2;16:919462. doi: 10.3389/fnins.2022.919462 (PMC9755855; doi:10.3389/fnins.2022.919462)

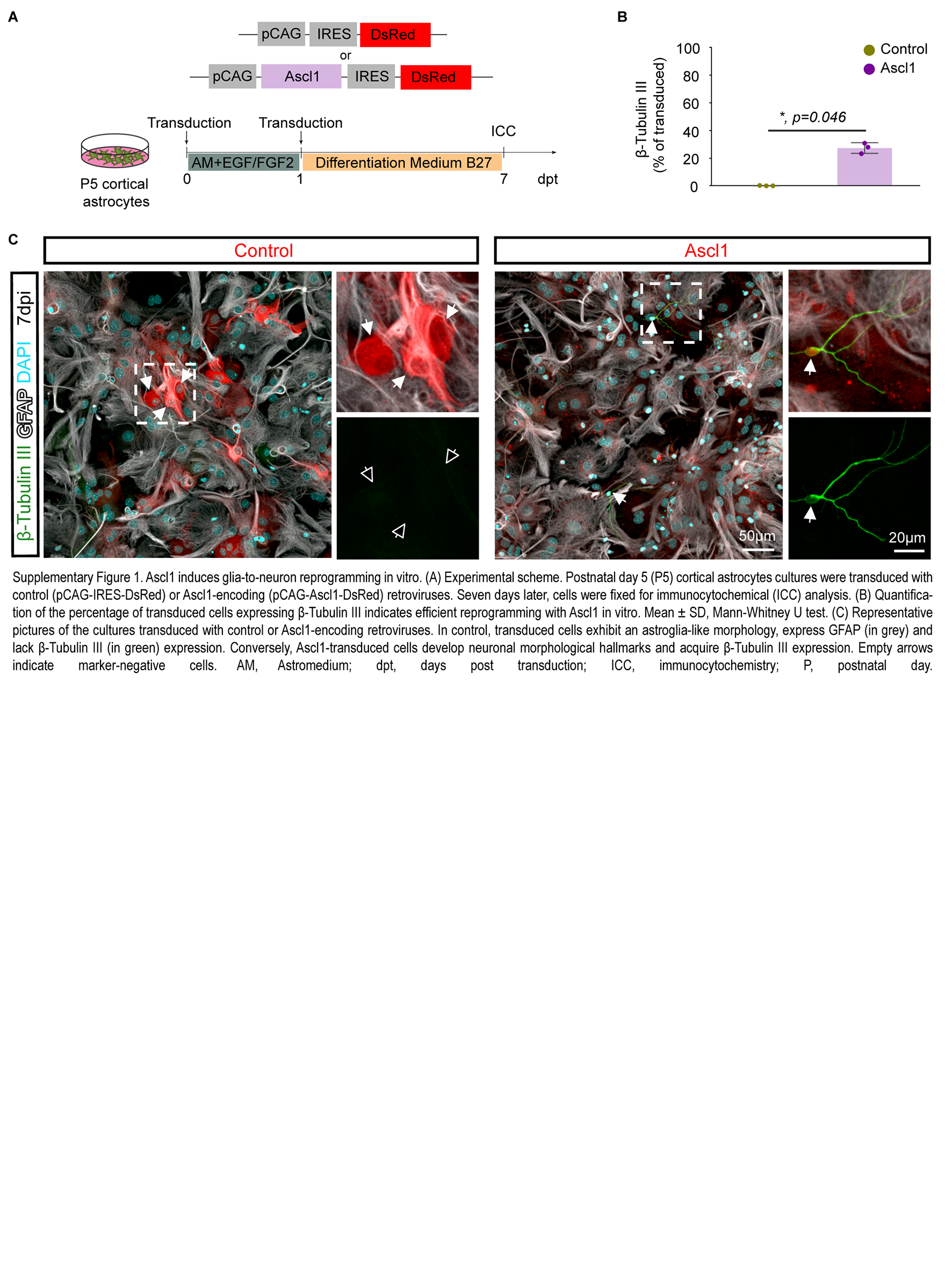

Supplement: Supplementary file 2 [file Image_1.TIF]
